# The Gut Microbiota in Parkinson’s Disease: Mechanistic Insights into Microbial–Host Interactions

**DOI:** 10.3390/microorganisms14030673

**Published:** 2026-03-16

**Authors:** Luis Enrique Guerrero-Torres, Jesús Jonathan García-Galindo, María Fernanda Gómez-Galindo, Diego Ian Rosales Delgado, Cesar Eduardo Retolaza Carlos, Daniel Osmar Suárez-Rico, Alberto Beltrán-Ramírez, Luis Ricardo Balleza Alejandri

**Affiliations:** 1 Centro Universitario de Ciencias de la Salud (CUCS), Universidad de Guadalajara, Guadalajara 44340, Mexico; luis.guerrero7543@alumnos.udg.mx (L.E.G.-T.); fernanda.gomez6625@alumnos.udg.mx (M.F.G.-G.); diego.rosales2026@alumnos.udg.mx (D.I.R.D.); 2 Departamento de Fisiología, Instituto de Terapéutica Experimental y Clínica, Centro Universitario de Ciencias de la Salud (CUCS), Universidad de Guadalajara, Guadalajara 44340, Mexico; jonathan.garcia@academicos.udg.mx (J.J.G.-G.); daniel.suarez@academicos.udg.mx (D.O.S.-R.); alberto.beltran@academicos.udg.mx (A.B.-R.); 3 Instituto de Terapéutica Experimental y Clínica (INTEC), Centro Universitario de Ciencias de la Salud (CUCS), Universidad de Guadalajara, Guadalajara 44340, Mexico; 4 Departamento de Neurocirugía, Antiguo Hospital Civil de Guadalajara “Fray Antonio Alcalde”, Guadalajara 44280, Mexico; cesar.retolaza7843@alumnos.udg.mx

**Keywords:** Parkinson’s disease, gut-brain axis, gut microbiota, gut dysbiosis, neuroinflammation, α-synuclein

## Abstract

Parkinson’s disease (PD) is a multifactorial neurodegenerative disorder characterized by progressive motor and non-motor manifestations, including early gastrointestinal dysfunction. Growing evidence implicates the gut microbiota as an active modulator of host immune tone and neurodegenerative vulnerability, extending beyond descriptive taxonomic associations toward functional and metabolic mechanisms. PD-associated dysbiosis is consistently characterized by altered microbial functional capacity, including reduced short-chain fatty acid (SCFA) production, enrichment of pro-inflammatory metabolic traits, and sustained immune stimulation at the intestinal interface. These shifts promote chronic low-grade inflammation and intestinal barrier perturbations, creating conditions that may facilitate abnormal α-synuclein aggregation within the enteric nervous system. Current management predominantly relies on dopaminergic replacement and related symptomatic strategies, such as levodopa combinations, dopamine agonists, monoamine oxidase-B and catechol-O-methyltransferase (COMT) inhibitors, and device-aided therapies, which alleviate symptoms but do not halt underlying neurodegeneration or modify long-term disease course. These therapeutic limitations have intensified interest in upstream mechanisms that might be amenable to disease-modifying interventions, particularly those arising at the level of the gut microbiota and gut–immune–brain axis. This narrative review integrates clinical, metagenomic, metabolomic, and mechanistic evidence to propose a unified model in which microbiota-driven immune and metabolic perturbations may act as upstream drivers converging on α-synuclein pathology, neuroinflammation, and neurovascular dysfunction.

## 1. Parkinson’s Disease

Parkinson’s disease (PD) is a progressive neurodegenerative disorder characterized by a heterogeneous constellation of motor and non-motor manifestations, reflecting multisystem involvement that extends beyond the classical dopaminergic nigrostriatal pathway [1,2]. PD affects millions of individuals worldwide, and its prevalence is expected to increase substantially due to population aging, representing a major public health challenge [3,4,5]. Beyond its epidemiological burden, the marked clinical heterogeneity of PD suggests that disease onset and progression are shaped by interacting central and peripheral processes rather than isolated neuronal vulnerability [1,3].

Clinically, the diagnosis is based on the presence of bradykinesia together with resting tremor and/or rigidity, whereas postural instability usually appears later in the course of the disease [3]. These symptoms are often initially asymmetric and derive mainly from dopaminergic dysfunction within the nigrostriatal system [2,3,6].

Pathologically, PD is defined by the progressive loss of dopaminergic neurons in the substantia nigra pars compacta and by the accumulation of misfolded α-synuclein in Lewy bodies and Lewy neurites throughout the central and peripheral nervous systems [3,7]. This degeneration results in dopamine depletion in the striatum, which is directly responsible for the classic motor symptoms of PD [2,4]. This misfolded α-synuclein serves not only as pathological markers but also as central mediators of disease propagation, leading to PD being classified as a synucleinopathy [4].

The Braak hypothesis proposes that in a subset of patients, the pathological process may begin in peripherally nervous structures, notably within the enteric nervous system (ENS) or olfactory bulb, and then ascends in a predictable sequence through the nervous system to involve higher cortical regions [2]. This propagation is believed to occur in a prion-like manner, possibly via the vagus nerve [2,4,6,7]. His staging is based on post-mortem neuropathological studies using α-synuclein immunostaining, which revealed a stereotyped progression of Lewy pathology from caudal to rostral brain regions [8].

However, the Braak hypothesis and its associated staging system do not account for all cases of Parkinson’s disease. Furthermore, the relationship between Braak stage and clinical severity is inconsistent, and not all individuals with Lewy pathology develop Parkinsonian symptoms [9,10], turning PD into a multisystem disorder that manifests a wide spectrum of non-motor symptoms [3]. It is important to note that many of these non-motor symptoms precede the onset of motor symptoms by several years, indicating the existence of a prodromal phase in the development of the disease [2,3,5,6]. This early multisystem involvement provides a critical framework for understanding subsequent neurodegenerative mechanisms.

Besides the neuronal pathology, immune system dysfunction and oxidative stress are increasingly implicated in PD-associated neuroinflammatory processes, contributing to sustaining microglial activation [2,7]. In this context, gut dysbiosis—characterized by altered microbial composition and function—has been correlated with increased exposure to microbial-derived inflammatory signals and elevated host inflammatory mediators, fostering a pro-inflammatory systemic environment [7].

PD is a complex, multifactorial disorder shaped by the interplay of demographic, genetic and environmental factors. Advanced age represents the most significant risk factor, with incidence steadily increasing throughout life [2,4,6]. Men are affected approximately 1.5 to 2 times more frequently than women, and ethnic variations have been described, with higher prevalence in populations of European descent [3,4]. Genetic predisposition is estimated to 22% to 40% of the total disease risk [2]. Well-defined monogenic forms include mutations in *PRKN*, *PINK1*, and DJ-1, which are associated with early-onset autosomal recessive PD, as well as in *SNCA*, which encodes α-synuclein itself. Variants in *GBA1* are associated with more aggressive disease phenotypes and an increased risk of dementia. In addition, more than 90 common susceptibility loci contribute to the polygenic architecture of sporadic PD [2,3,4,5].

Environmental and lifestyle factors further modulate disease risk. Exposure to pesticides, organic solvents, and heavy metals has been consistently associated with increased susceptibility to PD [4,6,7]. Additional risk modifiers include traumatic brain injury, dairy consumption, air pollution exposure, and type 2 diabetes mellitus [3,4]. Conversely, moderate caffeine consumption, regular physical activity, and the use of certain medications (e.g., Nonsteroidal Anti-Inflammatory Drugs (NSAIDs) and statins), appear to confer protective effects [3,7].

Current management of PD is primarily symptomatic and remains centered on dopaminergic replacement strategies. The cornerstone of therapy is levodopa, usually combined with a peripheral decarboxylase inhibitor, while dopamine agonists, monoamine oxidase-B inhibitors, COMT inhibitors, and other adjunctive agents are used to optimize motor control over the disease course. Although these regimens can markedly improve motor symptoms and quality of life, they do not prevent progressive neurodegeneration and are often limited by motor and non-motor complications, including dyskinesias and behavioral adverse effects. Advanced approaches such as deep brain stimulation and continuous dopaminergic infusion provide additional symptomatic benefit in selected patients but similarly lack proven disease-modifying effects. Thus, despite substantial progress in symptomatic care, there is still no established therapy that can slow or halt the underlying disease process, underscoring the need to identify upstream mechanisms—such as gut microbiota-driven immune and metabolic pathways—that might be amenable to preventive or disease-modifying interventions [3,7].

Collectively, these observations have shifted attention toward neuroinflammation and the gut–brain axis as integrative frameworks capable of linking peripheral immune disturbances with central neurodegeneration in PD [2,3,7].

## 2. Microbiota and the Gut–Brain Axis

The term “microbiota” refers to the diverse community of bacteria, archaea, fungi, and viruses that inhabit the human body, with the gastrointestinal tract hosting the largest and most complex ecosystem. In healthy adults, bacterial communities are dominated by *Firmicutes* and *Bacteroidetes*, alongside functionally relevant but less abundant phyla such as *Actinobacteria* and *Proteobacteria*, while the virome and mycobiota constitute additional integral components [11].

Initial microbial colonization occurs during and immediately after birth, in parallel with the development and formation of the host’s immune system. Unlike immune responses directed against pathogens, host–microbiota interactions induce immune programs that maintain a state of controlled inflammation and tolerance, commonly referred to as “homeostatic immunity”—a balance whose disruption is increasingly implicated in chronic inflammation and neurodegenerative conditions [12].

The human gut microbiota exhibits remarkable interindividual diversity, influenced by factors such as mode of delivery, diet, geographic location, age, sex, and lifestyle, yet broad compositional patterns—often referred to as “enterotypes”—can be described, typically enriched in *Bacteroides*, *Prevotella*, or SCFA-producing *Firmicutes* (including *Faecalibacterium* and *Ruminococcaceae* genera), with long-term dietary patterns acting as major determinants of these profiles [13,14].

Under conditions of eubiosis, the microbiota provides essential functions including nutrient metabolism, vitamin synthesis, colonization resistance against pathogens, and fine-tuning of mucosal and systemic immune homeostasis, which ultimately determine the interactions between the host and the microbiome [15].

Disruption of this balanced state, termed dysbiosis, involves ecological and functional changes such as expansion of pathobionts, loss of beneficial commensals, and altered metabolic output and has been linked to a wide range of metabolic, autoimmune, and neurodegenerative disorders [15].

In the context of the gut–brain axis—a bidirectional communication network integrating neural, immune, endocrine, and metabolic signaling—the microbiota exerts its effects through the production of neuroactive metabolites, modulation of barrier integrity, and regulation of immune and neural pathways that can influence central nervous system function. SCFAs are particularly important in this regard as they support intestinal and blood–brain barrier integrity, modulate systemic and microglial inflammation, and shape enteroendocrine and vagal signaling, positioning the gut microbiota as a key regulator of processes relevant to neurodegenerative diseases such as PD [16,17,18].

Several narrative and systematic reviews have described gut microbiota alterations in PD, primarily emphasizing compositional changes and taxonomic associations. Despite growing interest in the gut–brain axis in PD, the mechanistic integration of gut dysbiosis, immune activation, and neurodegeneration remains unresolved. Therefore, this narrative review aims to integrate and contextualize current clinical and experimental evidence on gut microbiota alterations in PD, with particular emphasis on their potential contribution to immune dysregulation, barrier dysfunction, and α-synuclein pathology within the gut–brain axis.

Conceptually, this review adopts a gut–immune–brain framework in which gut dysbiosis acts as an upstream trigger, immune amplification acts as a central driver, and CNS degeneration represents a downstream consequence, while acknowledging patient heterogeneity and alternative disease trajectories. 

First, the fundamental aspects of the pathophysiology of Parkinson’s disease and the physiological role of the gut microbiota in the gut–brain axis are described. Next, the key factors of gut dysbiosis, both environmental and host-related, are examined, and the microbiota alterations consistently reported in PD are summarized. Finally, the converging pathways linking gut dysbiosis to CNS involvement are analyzed, with particular emphasis on immune-mediated processes, barrier dysfunction, and α-synuclein pathology.

### 2.1. Microbiota and Its Role in the Development and Progression of Parkinson’s Disease

In PD, the gut microbiota maintains close communication with the CNS through multiple interconnected mechanisms, including vagal signaling, immune modulation, and the production of neuroactive metabolites such as SCFAs and neurotransmitters, including acetylcholine, serotonin, and dopamine. During states of dysbiosis, these communication pathways are disrupted, initiating local intestinal immune activation that can subsequently develop into chronic low-grade systemic inflammation. Consequently, gut dysbiosis has been linked to a wide spectrum of inflammatory and metabolic disorders, as well as neurodegenerative disorders, including PD [19].

Understanding the host-related and environmental factors that promote gut dysbiosis is essential for contextualizing its mechanistic contribution to the PD pathogenesis.

### 2.2. Factors That Cause Gut Dysbiosis

Gut dysbiosis is characterized by a series of ecological and functional changes within the gut microbial community. These typically include an expansion of potentially harmful bacteria, commonly referred to as pathobionts (resident microorganisms capable of exerting harmful effects under specific host or environmental conditions), a reduction in beneficial commensal microorganisms, and an overall decrease in microbial alpha-diversity. The factors driving dysbiosis can be broadly categorized as host-specific or environmental, both of which are highly relevant in the context of PD [20].

Among host-specific factors, genetic predisposition plays a key role by shaping intestinal niche characteristics that influence microbial colonization and persistence. Genetic variation at specific loci, such as LCT (lactase persistence and luminal lactose availability), NOD2 (innate immune sensing of microbial components), and FUT2 (regulation of mucin glycosylation and epithelial carbohydrate presentation), can directly influence the composition of the gut microbiome by selectively promoting or restricting the abundance of microbial species.

It is important to note that these genetically mediated effects do not act in isolation but interact dynamically with environmental factors, particularly diet. Dietary patterns can modulate, or in some contexts, override genetic predisposition, underscoring the substantial influence of environmental factors on gut microbial structure and function [21].

Age represents another critical determinant of microbial composition, particularly relevant for PD, given its strong association with aging. Advanced age is commonly associated with a reduction in beneficial symbionts and an expansion of opportunistic bacteria, a process often linked to age-related inflammation and an elevated basal inflammatory state. Furthermore, underlying inflammatory or infectious comorbidities can further disrupt the gut environment, favoring the proliferation of pathobionts [22].

Among environmental factors, antibiotic exposure represents one of the most potent disruptors of gut microbial ecology, as it can indiscriminately eliminate protective commensals and facilitate the overgrowth of opportunistic pathogens such as Clostridioides difficile. Paradoxically, certain antibiotics, particularly tetracycline derivatives such as doxycycline and minocycline, have demonstrated neuroprotective properties in models of neurodegenerative disease, including PD. These effects appear to be mediated not by antimicrobial activity but by direct anti-inflammatory, anti-apoptotic, and antioxidant actions that may mitigate mitochondrial dysfunction and microglial activation [23].

Lifestyle and diet exert a profound influence on gut microbial composition and function. High-fiber diets promote the expansion of beneficial, SCFA-producing bacteria, which supports epithelial integrity and immune homeostasis. In contrast, Western-style diets rich in fat and refined sugars reduce microbial diversity and increase the pro-inflammatory taxa. A lack of dietary diversity and excessive consumption of ultra-processed foods further contribute to the development of a pro-inflammatory gut environment [22].

Cumulatively and often synergistically, these host-related and environmental factors can lead to a persistent state of dysbiosis that compromises the host’s systemic health and may lower the threshold for neuroinflammatory processes relevant to PD.

A reduction in overall microbial alpha-diversity, often accompanied by a lower abundance of anti-inflammatory taxa and an enrichment of potentially pathogenic taxa, has been frequently reported in patients with PD. However, the nature and consistency of these changes vary substantially across cohorts; several studies have reported no significant differences compared with healthy controls, suggesting that reduced diversity may not represent a universal feature of PD-associated dysbiosis [19,24].

Analyses in drug-naïve PD cohorts, thereby minimizing medication-related confounding, have likewise failed to detect significant differences in alpha-diversity relative to lifestyle-matched controls, despite identifying multiple taxa with differential abundance. These findings support a model in which early dysbiosis in PD manifests primarily as compositional and functional remodeling rather than as a uniform loss of microbial diversity. Methodological variability, including differences in DNA extraction protocols, sequencing platforms, and bioinformatic pipelines, likely contributes to the heterogeneity across individual studies. Taken together, these data suggest that while reduced microbial diversity may be present in specific subgroups, it does not constitute a reproducible hallmark of PD [25].

In this context, a recent large-scale pooled analysis that harmonized methodological workflows across multiple cohorts found no systematic evidence for altered alpha-diversity in PD after controlling for technical and analytical biases [26].

### 2.3. Main Bacterial Taxa Decreased in Parkinson’s Disease

#### 2.3.1. *Lachnospiraceae* and *Ruminococcaceae* (e.g., *Blautia*, *Coprococcus*, *Roseburia*, *Faecalibacterium*, and *Ruminococcus*)

Metagenomic studies have consistently reported a reduced abundance of bacterial taxa with anti-inflammatory and barrier-supporting functions in patients with PD compared to healthy controls. Prominent among these are members of *Lachnospiraceae* and *Ruminococcaceae*, including the genera *Blautia*, *Coprococcus*, *Roseburia*, *Faecalibacterium*, and *Ruminococcus.* These taxa are major producers of butyrate, a key SCFA with pleiotropic effects on intestinal and immune homeostasis. Butyrate serves as a primary energy source for colonocytes and exerts broad regulatory effects on epithelial barrier integrity, immune tolerance and anti-inflammatory signaling, including the promotion of regulatory T cell differentiation [18,19,23,24].

#### 2.3.2. Genus *Prevotella*

The genus *Prevotella*, a Gram-negative member of the phylum *Bacteroidetes*, contributes to intestinal homeostasis through the degradation of complex dietary fibers and mucin glycans, supporting SCFA production. Reduced abundance of *Prevotella* has been associated with impaired mucin synthesis and turnover, potentially compromising the integrity of the intestinal mucus layer and increasing intestinal permeability, features frequently reported in PD [19,24]. In addition, *Prevotella* depletion has been associated with alterations in ghrelin secretion and signaling. Ghrelin, a peptide hormone primarily produced by the stomach and small intestine, exerts central effects through receptors expressed in multiple brain regions, including the substantia nigra. Although its precise role in PD remains incompletely understood, accumulating evidence suggests that ghrelin may exert neuroprotective effects on the nigrostriatal dopaminergic pathway [18,27].

Taken together, the consistent depletion of *Lachnospiraceae*, *Ruminococcaceae*, and *Prevotella*-related taxa in PD points toward a shared functional deficit—rather than isolated taxonomic losses—centered on reduced fermentative capacity, impaired barrier support and diminished immunoregulatory signaling. These taxa converge in their ability to ferment complex carbohydrates and mucins, producing SCFA, particularly butyrate, that support epithelial barrier integrity and immune tolerance Their reduction suggests a shift toward a gut microbial ecosystem with diminished functional resilience, potentially lowering the threshold for intestinal permeability and immune activation. However, whether this functional impairment represents a primary pathogenic event or a secondary consequence of disease-related factors, such as dietary changes, medication exposure, or altered gut motility, remains unresolved.

#### 2.3.3. *Lactobacillaceae* and *Bifidobacteriaceae*: Context-Dependent Enrichment in Parkinson’s Disease

Although often regarded as beneficial in certain contexts due to their production of lactate and acetate, competitive exclusion of pathogens, and bacteriocin synthesis, increased abundance of *Lactobacillaceae* and *Bifidobacteriaceae* has been consistently reported in PD. This enrichment may reflect two non-mutually exclusive mechanisms rather than a direct protective effect. First, correlations have been observed between increased abundance of these taxa and clinical indicators of inflammation, such as elevated neutrophil and monocyte counts, suggesting a compensatory microbial response aimed at restoring intestinal homeostasis. Second, antiparkinsonian medications, particularly COMT inhibitors, may directly or indirectly modulate microbial growth, favoring these taxa [18,19,23,24].

A recurrent observation across cohorts is the increased abundance of *Lactobacillaceae* in PD; however, interpretation of this signal requires species-level and functional resolution. Narrative synthesis highlights an apparent paradox: some *Lactobacillaceae* species have been correlated with worse motor function and may reduce levodopa bioavailability through tyrosine decarboxylase (TDC) activity, whereas other strains, often administered as probiotics, have been associated with improvements in gastrointestinal symptoms and quality of life. Importantly, the direction of causality remains unresolved, and prolonged levodopa exposure has been hypothesized to select for TDC-producing taxa, illustrating the bidirectional coupling between antiparkinsonian therapy and microbial ecology [28].

#### 2.3.4. Genus *Akkermansia*

Species of the mucin-degrading genus *Akkermansia* are frequently enriched in patients with PD (24). While *Akkermansia* has been associated with metabolic health in some contexts, its role in PD appears to be highly context-dependent. Under conditions of excessive abundance, its reliance on mucin as a primary energy source may contribute to intestinal hyperpermeability (“leaky gut”) by accelerating degradation of the protective mucus layer, thereby increasing host exposure to luminal antigens [19,27,29,30].

#### 2.3.5. Phylum Proteobacteria

Expansion of Proteobacteria represents a common signature of dysbiosis in PD. This includes genera such as *Escherichia/Shigella*, which produce lipopolysaccharides (LPS), and other pro-inflammatory molecules. These microbial products have been implicated in processes that may compromise striatal microvascular and astrocytic integrity, potentially contributing to blood–brain barrier dysfunction, neuroinflammation, and neural vulnerability. Other Proteobacteria frequently reported to be increased in PD include *Acinetobacter*, *Bilophila*, *Enterobacter*, and *Klebsiella* [24].

#### 2.3.6. Specific *Firmicutes* and *Helicobacteraceae*

While the phylum Firmicutes dominate, specific genera such as *Enterococcus*, *Streptococcus*, and *Christensenella* are often enriched in PD. Some of these taxa can produce endotoxins and other pro-inflammatory mediators that may contribute to sustained immune activation [24]. In addition, infection with *Helicobacter pylori* is more prevalent in PD and has been associated with impaired levodopa absorption and heightened peripheral immune responses involving NK and CD4+ T cells, correlating with worse motor symptoms. Proposed mechanisms include neurotoxic effects mediated by bacterial cholesterol glycosides that damage dopaminergic neurons and potential translocation across the compromised blood–brain barrier, where it might directly induce apoptosis of dopaminergic neurons, as well as compromised levodopa bioavailability [19,30].

Beyond these commonly reported genera, a large harmonized meta-analysis has identified additional taxa that are consistently elevated in PD, including members of the phylum *Firmicutes* and the genera *Eisenbergiella*, *Desulfurispora*, and *Acidaminobacter*. While their lower abundance has made them less prominent in individual studies, their consistent identification in pooled analyses suggests a potential role in the PD gut environment that warrants further investigation [26].

**Figure 1:**
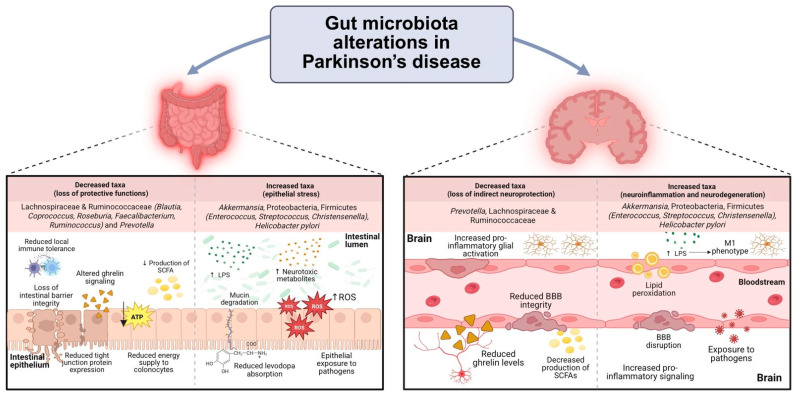
Functional consequences of gut microbiota alterations in Parkinson’s disease across the gut–brain axis. Schematic representation of consistent taxonomic shifts reported in PD and their associated implications at the intestinal and CNS levels. Reduced abundance of SCFA-producing taxa, including *Lachnospiraceae*, *Ruminococcaceae*, and *Prevotella*, is associated with impaired epithelial barrier integrity, reduced immune tolerance, altered ghrelin signaling, and decreased energy supply to colonocytes. In contrast, increased abundance of taxa such as *Akkermansia*, *Proteobacteria*, and specific Firmicutes is linked to mucin degradation, elevated LPS exposure, oxidative stress, and sustained immune activation. These intestinal alterations may promote systemic inflammation, compromise blood–brain barrier integrity, and facilitate microglial activation, thereby contributing to neuroinflammatory signaling and increased neurodegenerative vulnerability. Importantly, the figure emphasizes functional convergence rather than isolated taxonomic changes, highlighting the context-dependent nature of microbial enrichment in Parkinson’s disease. Created with BioRender. Gómez, F. (2026) https://BioRender.com/jzukcqh (accessed on 3 February 2026).

Collectively, these compositional shifts are consistent with a gut microbial ecosystem enriched in pro-inflammatory, mucin-degrading and barrier-disruptive functional traits (Table 1; Figure 1). Importantly, taxonomic enrichment alone does not equate to biological activity, underscoring the need for functional and strain-level validation to clarify whether these changes represent compensatory responses, medication-driven niche selection, or contributors to PD pathogenesis.

From a mechanistic perspective, the functional outcomes of the gut microbiome, rather than taxonomic composition per se, represent the most biologically plausible interface through which gut dysbiosis can influence host immune tone and subsequent neurodegenerative vulnerability.

Beyond compositional features, the metagenomic profile indicates that PD is associated with reproducible functional reprogramming of the gut ecosystem. In a longitudinally phenotyped cohort, baseline metagenomics identified disease-associated taxonomic and functional changes, with a subset of microbial functional terms correlated with subsequent clinical progression, as measured by the Movement Disorder Society-Unified PD Rating Scale (MDS-UPDRS). Notably, these functional signatures distinguished slower-progressing patients from faster-progressing ones with moderate discriminatory performance and could be clinically informative, possibly aligning with proposed gut-first versus brain-first disease trajectories [31].

Prospective metabolic analyses further support a temporal association between microbiota-linked metabolic signatures and future PD risk, strengthening the biological plausibility of microbiome–host metabolic pathways contributing to disease susceptibility [32]. However, despite longitudinal correlations suggesting biological relevance, most available evidence remains observational and susceptible to residual confounding.

In this context, genetics-anchored approaches provide an additional layer of inference. Using Mendelian randomization, Yan and Zhao reported largely non-robust causal signals for individual gut taxa after multiple testing correction, whereas higher circulating levels of branched-chain amino acids (particularly isoleucine) were associated with reduced PD risk following false discovery rate adjustment [33]. Although constrained by taxonomic resolution and instrument strength, these findings reinforce the concept that microbiota-associated metabolic pathways, rather than taxonomic changes alone, may represent more biologically informative intermediates linking gut ecosystem alterations to disease susceptibility and subsequent neurodegenerative mechanisms.

## 3. Abnormal Aggregation of α-Synuclein

Abnormal α-synuclein aggregation represents a crucial mechanistic link between gut dysbiosis, immune activation, and central neurodegeneration, rather than merely an isolated neuropathological feature.

**Figure 2:**
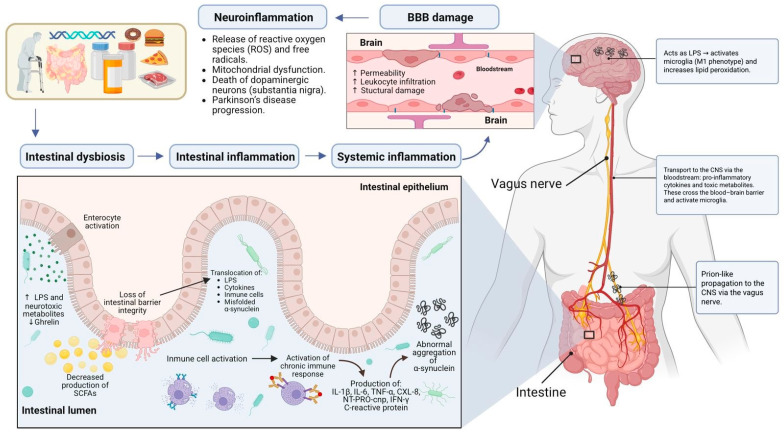
Mechanisms linking gut dysbiosis to PD pathogenesis. Gut dysbiosis, characterized by a loss of SCFA-producing taxa (e.g., *Faecalibacterium*, *Roseburia*, and *Prevotella*) and an increase in pathobiont species (e.g., *Akkermansia*, *Proteobacteria*, and *Helicobacter* pylori), leads to intestinal barrier dysfunction (“leaky gut”) and innate/adaptive immune activation. Barrier dysfunction facilitates the translocation of microbial-associated molecular patterns (MAMPs), including lipopolysaccharide (LPS), and the release of pro-inflammatory cytokines (e.g., IL-1β, IL-6, TNF-α, and IFN-γ) into the lamina propia and systemic circulation. In parallel, diminished SCFA signaling and persistent inflammation contribute to blood–brain barrier (BBB) disruption via mechanisms involving oxidative stress, activation of the NLRP3 inflammasome, and signaling through Toll-like receptors (TLRs). Concurrently, within the ENS, dysbiosis promotes α-synuclein misfolding and aggregation. These pathological aggregates can then spread retrogradely to the CNS via the vagus nerve. In the brain, this process is associated with microglial activation (predominantly the pro-inflammatory M1-like phenotype), chronic neuroinflammation, and ultimately the death of dopaminergic neurons in the substantia nigra pars compacta. Created with BioRender. Gómez, F. (2026) https://BioRender.com/9chp6s7 (accessed on 3 February 2026).

It is proposed that gut dysbiosis may promote the initial misfolding and aggregation of the α-synuclein within the ENS [34]. It is noteworthy that pathological α-synuclein has been detected in the ENS prior to its appearance in the CNS, supporting the hypothesis of a prion-like propagation from the gut to the brain. According to this model, pathological α-synuclein aggregates can propagate retrogradely along the vagus nerve via neuronal junction, uptake, and internalization of preformed fibrils. It has been hypothesized that the aggregates first reach the dorsal motor nucleus of the vagus (DMV) and the locus coeruleus. From there, it is proposed that they ascend through the medulla oblongata and eventually reach the substantia nigra pars compacta, a key site of dopaminergic neurodegeneration in PD (Figure 2) [23,29,33]. As mentioned above, this Braak staging hypothesis is supported by postmortem findings showing α-synuclein pathology in the DMV of early-stage PD cases. Additionally, preliminary epidemiological evidence indicates that complete truncal vagotomy may be associated with a modestly reduced risk of developing PD [35]. The detection of pathological α-synuclein, not only in the gastrointestinal tract but also in other organs with vagal innervation, such as the heart, further supports this hypothesis [23].

Under physiological conditions, α-synuclein exists primarily as an intrinsically disordered, soluble monomer involved in synaptic vesicle recycling and neurotransmission. Under pathological conditions, α-synuclein can misfold into β-sheet-rich conformations, forming oligomers and ultimately mature fibrils that aggregate into Lewy bodies and Lewy neurites, the characteristic pathological inclusions of PD. This process can be accelerated by multiple factors, including exposure to environmental toxins, oxidative stress, mitochondrial dysfunction, and specific genetic mutations (e.g., in the SNCA or GBA1 genes) [29,33].

Beyond host-derived stressors, dysbiosis may introduce microbial structural signals that interact with protein misfolding. Bacterial amyloid-like proteins, such as curli fibers, and endotoxin-associated inflammatory signaling have been proposed as potential facilitators of α-synuclein misfolding and aggregation, either by providing cross-seeding-like stimuli or by creating a permissive pro-inflammatory environment that lowers the threshold for pathological proteostasis. While these models remain largely inferential, they provide a microbiologically grounded rationale for how distinct dysbiotic states might converge into a shared α-synuclein-centered pathology [36].

Elevated levels of abnormally aggregated α-synuclein can contribute to oxidative stress by increasing lipid peroxidation in dopaminergic neurons pro-inflammatory, leading to an increase in reactive oxygen and nitrogen species [36,37].

Furthermore, chronic exposure to α-synuclein aggregates, especially in their fibrillar form, and in combination with inflammatory signals such as TNF-α and PGE2, can polarize microglia toward an inflammatory phenotype with metabolic and functional characteristics similar to classical M1, inducing robust activation of microglia and monocytes, promoting the release of other pro-inflammatory cytokines (e.g., IL-1β) and the expression of markers associated with this phenotype, but with particularities such as increased glutamate release and alterations in iron and glutathione metabolism, which increase neurotoxicity [37].

It is worth nothing that misfolded α-synuclein has been described to exhibit PAMP-like immunostimulatory properties (LPS analogs) from Gram-negative bacteria [36,37]. Like LPS, it can be recognized by pattern recognition receptors (PRRs) on innate immune cells, promoting innate immunity activation [37,38]. This molecular mimicry allows misfolded α-synuclein to induce robust microglial activation and promote a shift toward an M1-like neurotoxic state, thereby contributing to a chronic neuroinflammatory environment [38]. Consequently, activated microglia releases pro-inflammatory cytokines (e.g., TNF-α, and IL-1β), and reactive oxygen species (ROS), which collectively contribute to neuronal injury, synaptic dysfunction, and, ultimately, disease progression [37,39,40].

Emerging evidence indicates that adaptive immunity, particularly CD4+ T cell-mediated responses, also contributes to the inflammatory cascade observed in PD [7,41]. Signals derived from the dysbiotic states (such as microbial metabolites, structural components, and microbe-derived neuroactive compounds) may initiate or exacerbate this response. Furthermore, molecular mimicry may allow microbial antigens to share epitopes with autoantigens, such as the misfolded α-synuclein present in Lewy bodies [36,41].

In either scenario, activated CD4+ T cells can differentiate into pro-inflammatory T helper 1 and 17 (Th 1 and Th17). These cells then release distinctive cytokines such as interferon-gamma (IFN-γ) and interleukin-17 (IL-17), which can recruit and activate other immune cells (e.g., macrophages and neutrophils), potentially perpetuating a state of chronic inflammation, initially within the intestinal mucosa [41,42].

The cytokines and inflammatory environment generated by Th1 and Th17 responses can, in turn, further promote α-synuclein misfolding and aggregation within the enteric nervous system, creating a vicious cycle that favors its propagation toward the CNS [33]. Consistent with broader neurodegeneration frameworks, microbiota–immune interactions in PD have been analyzed in terms of adaptive shift toward pro-inflammatory helper T cell, including those mentioned above. These responses can act as systemic amplifiers linking mucosal immune activation with subsequent CNS inflammatory susceptibility [41].

In support of these mechanistic links, reports of early α-synuclein pathology in the ENS and its proposed role in altered intestinal motility could support its clinical relevance as a key prodromal feature of the disease, as it may contribute to the development of constipation, a common early non-motor symptom in PD, which frequently precedes the motor diagnosis by several years [37,43]. Furthermore, evidence from colon biopsies in PD patients reveals increased infiltration of immune cells and elevated levels of pro-inflammatory cytokines, directly supporting local inflammatory processes that coincide with α-synuclein pathology in PD [37,43]. This clinical observation aligns with reports of early α-synuclein pathology in the ENS and its proposed role in disrupting gut motility, supporting its relevance as a key prodromal feature of the disease [37]. Furthermore, evidence from colon biopsies of PD patients reveals increased immune cell infiltration and elevated levels of pro-inflammatory cytokines, providing direct support for local inflammatory processes coinciding with α-synuclein pathology [33,43].

Taken together, these observations support a consistent sequence in which intestinal α-synuclein pathology coexists with local immune activation and barrier disruption, thus establishing a plausible pathway for systemic inflammatory signaling with relevance to the CNS.

## 4. Systemic Inflammation and Neuroinflammation Induced by Dysbiosis

There is robust evidence and increasing evidence that gut dysbiosis is viewed as a plausible upstream driver of low-grade chronic systemic inflammation, a feature observed in a substantial proportion of PD patients [23,43]. In parallel, early-onset PD has been associated with increased intestinal permeability, with elevated markers of intestinal barrier dysfunction such as zonulin and calprotectin, compared to healthy controls, and intestinal inflammation [44,45]. These findings are supported by histological evidence of reduced expression of tight junction proteins (e.g., ZO-1 and claudin-1) and mucosal remodeling in colonic biopsies from PD patients, indicating compromised epithelial barrier integrity [23,43,46].

These barrier alterations and associated inflammatory changes may facilitate the translocation of pro-inflammatory signals into the systemic circulation, including microbial components (e.g., LPS), microbially derived metabolites, and other pro-inflammatory molecules, which can perpetuate and amplify the systemic inflammatory response [19,43,46,47].

It is also important to consider dietary habits, as environmental toxins can promote systemic inflammation and mitochondrial stress, as well as drive neuroinflammatory signaling. In this context, the neurotoxin β-methylamino-L-alanine (BMAA), a non-proteinogenic amino acid produced by cyanobacteria, diatoms, and dinoflagellates, is found in various environmental sources, such as shellfish and contaminated water, rather than microbiota-derived production [48]. There is increasing evidence, derived from experimental and epidemiological studies, that exposure to BMAA may contribute to the development and progression of Parkinson’s disease (PD) through several mechanisms linked to microglial activation. In vitro studies have shown that chronic dietary exposure to BMAA can induce intestinal dysbiosis, increase intestinal inflammation, impair barrier integrity, and promote caudorostral progression of α-synuclein aggregation, which is consistent with the “gut-first” hypothesis in PD pathogenesis (Figure 2) [19,23,24,43].

From a mechanistic perspective, increased intestinal permeability in PD can be conceptualized as a failure of a multilayer barrier system, rather than a single “tight junction-defect” [44,49]. The intestinal barrier integrates the mucus layer, epithelial integrity, and junctional complexes (e.g., occludin and claudins) coordinated with mucosal immune surveillance. In dysbiosis, decreased barrier support functions and increased pro-inflammatory signals can weaken this architecture, facilitating the passage of microbe-associated molecular patterns and other luminal signals into circulation. Consequently, leaky gut becomes a potential amplifier of systemic inflammation, rather than a purely local gastrointestinal finding [49].

Key inflammatory mediators elevated in PD include pro-inflammatory cytokines (e.g., IL-1β, IL-6, TNF-α, and IFN-γ), chemokines (e.g., CXCL8/IL-8), and acute-phase proteins (e.g., C-reactive protein), which reflects not only ongoing intestinal and systemic immune activation but also potentially exerting distal effects on the nervous system [33,42,43,46]. Some of these circulating inflammatory mediators can directly cross a compromised BBB, while others bind receptors on cerebral endothelial cells, further increasing BBB permeability [37]. This facilitates immune cell recruitment and amplifies microglial activation, promoting neuroinflammation, oxidative stress, and mitochondrial dysfunction, and creating an environment that can enhance the aggregation intercellular spread of pathological α-synuclein in dopaminergic neurons of the substantia nigra, which constitute established pathways of neuronal injury in PD [33,42,46].

In this way, systemic inflammation functions not only as a correlate of gut pathology but also as a mechanistic conduit linking intestinal barrier failure with neurovascular unit instability and innate immune activation of the CNS.

## 5. Blood–Brain Barrier Dysfunction and Microglial Activation: The Brain-First Model

The blood–brain barrier is a highly dynamic and selective interface that protects the CNS from potentially harmful circulating agents. Its functional integrity is maintained by the neurovascular unit (NVU), a multicellular complex composed of cerebral endothelial cells, pericytes, astrocytes, and the basement membrane [50].

In the “brain-first” model of PD pathogenesis, neurodegeneration is thought to originate within the central nervous system, particularly in the substantia nigra, rather than being initiated by peripheral or gut-derived factors. In this context, BBB dysfunction and microglial activation are central, interacting drivers of disease progression [51].

Microglial activation occurs early in PD and should be conceptualized not only as a downstream response to BBB disruption, but also as an active contributor to neurovascular dysfunction and is a key mediator of neuroinflammation and dopaminergic neuronal injury. In this context, microglia represent a critical cellular interface through which systemic inflammatory signals may be translated into sustained CNS immune activation and neurovascular instability [52].

Activated microglia release pro-inflammatory cytokines and matrix metalloproteinases (notably MMP-2 and MMP-9), which degrade tight junction proteins (e.g., ZO-1, claudin-5, and occludin), leading to increased BBB permeability and disruption of barrier integrity. Once the BBB is compromised, the influx of cytokines and peripheral immune cells into the brain parenchyma triggers the activation of microglia, which often adopts a phagocytic phenotype, which can release further inflammatory mediators that, in a vicious cycle, further increase BBB permeability [39,53].

This pattern aligns with the concept of a self-sustained neurodegeneration–neuroinflammation loop in PD: once innate immune activation becomes established, inflammatory effector programs can both respond to and exacerbate neuronal stress, creating a feed-forward circuit that helps explain persistence and progression even when the initiating trigger is no longer dominant [40]. The resulting environment promotes α-synuclein aggregation, mitochondrial dysfunction, and progressive dopaminergic neurodegeneration. within the striatum, a primary target in PD [54].

Additionally, astrocytic dysfunction and inflammatory signaling further compromise BBB integrity, exacerbating the cycle of neuroinflammation and neuronal injury. Notably, the BBB within the striatum (a primary target in PD) appears to be particularly vulnerable to this disruption [55].

Imaging and experimental studies confirm that BBB breakdown is not merely a consequence of neuronal loss but actively contributes to disease propagation by disturbing the neural microenvironment and facilitating the entry of peripheral inflammatory mediators alongside producing additional chemokines (e.g., CCL2). This BBB dysfunction allows peripheral immune cells, iron, and other neurotoxic blood-derived factors to enter the brain parenchyma, further amplifying microglial activation and neuroinflammation in a feed-forward loop, exacerbating neuroinflammation [18,42,54,55].

## 6. Parallel Dual-Origin Model of PD: When the Gut and Brain Meet

Building upon the systemic inflammatory environment described above, the BBB represents a critical and highly dynamic interface through which peripheral immune and metabolic disturbances can gain access to the CNS [42].

Chronic systemic inflammation, driven by gut dysbiosis, can partially compromise the integrity of the BBB. Circulating pro-inflammatory cytokines mediate this detrimental effect through distinct mechanisms: IL-6 downregulates key tight-junction proteins such as claudin-5 and occludin; TNF-α increases endothelial permeability and disrupts the protective glycocalyx layer; and IL-17, IL-22, and IFN-γ synergistically impair junctional integrity [23,42,46]. Rather than representing a passive structural failure, BBB dysfunction in this context reflects an active, inflammation-driven process that sensitizes the neurovascular unit to peripheral immune signals.

Furthermore, microbially derived factors can directly contribute to BBB disruption. An increase in LPS-containing bacteria (e.g., *Enterobacteriaceae*) provides a persistent source of TLR4 agonists. Concurrently, a decrease in SCFA-producing bacteria (e.g., *Prevotella* and *Faecalibacterium*) leads to a deficit of these beneficial metabolites, which normally help maintain the integrity of BBB and exert anti-inflammatory effects [56]. This dual assault—an excess of triggers and loss of protective factors—promotes oxidative stress, mitochondrial dysfunction, and activation of innate immune pathways like the NLRP3 inflammasome and TLR signaling (particularly TLR2 and TLR4), thereby driving neuroinflammation and contributing to neurodegenerative processes [34,49].

This self-perpetuating cycle of microglial activation, increased BBB permeability, and subsequent neuroinflammation, initially driven by peripheral immune perturbations, constitutes a core pathophysiological mechanism underpinning neurodegeneration in PD. A similar process, often exacerbated by gut dysbiosis, is also implicated in brain aging, suggesting shared pathways between age-related vulnerability and disease-specific pathogenesis [18,46].

## 7. Therapeutic Implications and Microbiota-Targeted Interventions

The emerging recognition of gut dysbiosis as a potential contributor to PD has prompted interest in microbiota-targeted strategies as adjuncts to conventional dopaminergic therapies. Rather than focusing on individual taxa, these approaches aim to restore barrier integrity, dampen chronic low-grade inflammation, and normalize microbial metabolic outputs such as SCFA and other neuroactive metabolites. Conceptually, dietary interventions enriched in fermentable fibers, as well as selected prebiotic and probiotic formulations, may promote the expansion of beneficial commensals and counteract the enrichment of pro-inflammatory, mucin-degrading, or pathobiont populations described in Parkinson’s disease [57,58].

Early experimental and clinical studies suggest that microbiome-modulating strategies can influence gut inflammation, barrier function, and, in some cases, motor or non-motor symptoms, but the available evidence remains preliminary and heterogeneous [59]. Larger, controlled trials with standardized microbiome and clinical endpoints are required to determine which modalities, timing, and patient subgroups are most likely to benefit and to ensure long-term safety. At present, microbiota-targeted interventions should therefore be viewed as promising but still investigational tools that complement, rather than replace, established symptomatic treatments for Parkinson’s disease [60,61].

## 8. Future Directions

Future research on the gut–immune–brain axis in PD should prioritize longitudinal and mechanistic designs capable of disentangling cause from consequence in microbiota alterations. Large, deeply phenotyped cohorts integrating metagenomics, metabolomics, immune profiling, and high-resolution clinical trajectories are needed to determine whether specific microbial functions or metabolites precede disease onset, track with progression, or define distinct gut-first and brain-first trajectories. Harmonized methodologies and robust control for confounders such as diet, medications, and comorbidities will be essential to increase reproducibility and to identify microbiome-derived biomarkers that can be translated into risk stratification or early diagnosis [61].

Experimental work should further delineate how discrete microbial metabolites, immune pathways, and barrier changes converge on α-synuclein pathology and neuroinflammation in vulnerable neural circuits. Combining gnotobiotic models, humanized microbiota transfers, and in vitro systems with patient-derived samples may help to validate candidate mechanisms and to identify microbial signatures with true causal relevance. Finally, carefully designed interventional trials—ranging from diet-based and prebiotic strategies to targeted microbial consortia or other microbiota-modulating approaches—will be required to test whether modifying the gut ecosystem can meaningfully alter clinical outcomes or biological markers in Parkinson’s disease [59].

## 9. Conclusions

Accumulated clinical and experimental evidence supports the fundamental role of the gut microbiota in the pathogenesis and progression of PD, positioning gut dysbiosis not as an isolated gastrointestinal feature but as a modulator of systemic, neurovascular, and immune processes relevant to neurodegeneration.

Dysbiosis-associated loss of barrier-supportive functions and enrichment of pro-inflammatory microbial signals converge to increase intestinal permeability, facilitating systemic exposure to microbial components, metabolites and inflammatory mediators. This chronic inflammatory state may sensitize the neurovascular unit, compromise the BBB integrity, and promote sustained microglial activation. In parallel, immune distortion and microbial-derived signals associated with dysbiosis may lower the threshold for abnormal α-synuclein folding and aggregation within the ENS, reinforcing neuroinflammatory cascades and gut–brain propagation pathways.

Taken together, these processes are best conceptualized as a self-reinforcing feedback loop that links intestinal barrier dysfunction, systemic inflammation, neurovascular instability, and innate immune activation. While much of the current evidence remains associative and derived from heterogeneous models, this integrative framework highlights the gut microbiota as a promising target for disease-modifying strategies aimed at disrupting pathogenic immune–neurovascular feedback loops. These insights reinforce the view of PD as a systemic disorder and support continued investigation of microbiome-centered approaches beyond symptomatic treatment, with therapeutic strategies targeting the gut–brain axis—including probiotics, prebiotics, dietary interventions, antibiotics, and fecal microbiota transplantation.

## Figures and Tables

**Table 1:** Summary of major microbiota-related mechanisms along the gut–brain axis in PD and their proposed implications.

Microbial Alteration/Mechanism	Representative Taxa or Signals	Main Microbial–Host Pathways Involved	Proposed Implications for Parkinson’s Disease
Reduced SCFA-producing commensals	Families *Lachnospiraceae* and *Ruminococcaceae* (e.g., *Blautia*, *Coprococcus*, *Roseburia*, *Faecalibacterium*, *Ruminococcus*), genus *Prevotella*	Decreased fermentation of complex carbohydrates and mucins, reduced butyrate production, impaired support of epithelial barrier integrity and regulatory T cell-mediated immune tolerance	Favors intestinal permeability and a shift toward a pro-inflammatory milieu, lowering the threshold for chronic low-grade inflammation and facilitating α-synuclein aggregation within the enteric nervous system
Enrichment of mucin-degrading bacteria	Genus *Akkermansia*	Accelerated degradation of the mucus layer, increased exposure of epithelium to luminal antigens and microbial products	Contributes to “leaky gut,” enhancing translocation of microbial-associated molecular patterns and promoting local and systemic immune activation in PD
Expansion of Proteobacteria and other pathobionts	Phylum Proteobacteria (e.g., *Escherichia*/*Shigella, Acinetobacter*, *Bilophila, Enterobacter*, *Klebsiella*), increased prevalence of *Helicobacter pylori*	Enhanced production of lipopolysaccharide and other pro-inflammatory mediators, oxidative stress, and endothelial activation	Drives systemic inflammation, may contribute to blood–brain barrier dysfunction and has been associated with worse motor symptoms and altered levodopa pharmacokinetics in PD
Context-dependent enrichment of Lactobacillaceae and Bifidobacteriaceae	Families Lactobacillaceae and Bifidobacteriaceae	Production of lactate and acetate, competition with other taxa, potential interaction with antiparkinsonian medications (e.g., COMT inhibitors) and tyrosine decarboxylase activity	May reflect a compensatory response to inflammation or medication-driven niche selection; certain strains could influence levodopa bioavailability and gastrointestinal symptoms, but causal direction and net impact remain uncertain
Chronic intestinal and systemic immune activation	Translocation of MAMPs (e.g., LPS), increased cytokines such as IL-1β, IL-6, TNF-α, IFN-γ	Activation of innate and adaptive immune pathways in the lamina propria and periphery, NLRP3 inflammasome signaling, Toll-like receptor engagement	Establishes persistent low-grade inflammation, primes circulating immune cells, and promotes microglial sensitization to subsequent inflammatory or neurotoxic stimuli in the CNS
Barrier dysfunction in gut and blood–brain interfaces	Reduced SCFA signaling, oxidative stress, pro-inflammatory mediators	Disruption of tight junctions in the intestinal epithelium and blood–brain barrier, altered endothelial and astrocytic function	Facilitates systemic dissemination of microbial products and inflammatory mediators, thereby enhancing neurovascular vulnerability and access of peripheral signals to the brain parenchyma
α-Synuclein misfolding and propagation from the gut	Pathological α-synuclein aggregates in the enteric nervous system; bacterial amyloid-like proteins (e.g., curli) and endotoxin-driven inflammation	Cross-seeding-like interactions, prion-like spread of α-synuclein along the vagus nerve from the ENS to the dorsal motor nucleus of the vagus and midbrain nuclei	Links gut dysbiosis to early α-synuclein pathology and its rostro-caudal propagation, providing a mechanistic bridge between peripheral inflammation and nigrostriatal neurodegeneration in PD
Neuroinflammation and dopaminergic neuron loss	Fibrillar α-synuclein, pro-inflammatory cytokines, microglial M1-like polarization	Microglial and monocyte activation, increased production of reactive oxygen and nitrogen species, alterations in glutamate, iron, and glutathione metabolism	Sustained neuroinflammatory circuits and oxidative stress culminate in degeneration of dopaminergic neurons in the substantia nigra pars compacta, driving progression of motor and non-motor symptoms in PD

This table summarizes key patterns of gut dysbiosis reported in Parkinson’s disease, the principal microbial–host interaction pathways they engage, and their proposed relevance for α-synuclein pathology, barrier disruption, systemic inflammation, and neurodegeneration along the gut–immune–brain axis.

## Data Availability

No new data were created or analyzed in this study. Data sharing is therefore not applicable to this article.

## Abbreviations

The following abbreviations are used in this manuscript:

BBB	Blood–brain barrier
BMAA	β-Methylamino-L-alanine
CNS	Central nervous system
COMT	Catechol-O-methyltransferase
CRP	C-reactive protein
CXCL8	C-X-C motif chemokine ligand 8
DMV	Dorsal motor nucleus of the vagus
ENS	Enteric nervous system
GBA1	Glucocerebrosidase gene
GLP-1	Glucagon-like peptide-1
IFN-γ	Interferon gamma
IL-1β	Interleukin-1 beta
IL-6	Interleukin-6
IL-8	Interleukin-8
IL-17	Interleukin-17
IL-22	Interleukin-22
LPS	Lipopolysaccharide
MDS-UPDRS	Movement Disorder Society–Unified Parkinson’s disease Rating Scale
NK cells	Natural killer cells
NLRP3	NLR family pyrin domain containing 3
NSAIDs	Nonsteroidal anti-inflammatory drugs
NVU	Neurovascular unit
PAMPs	Pathogen-associated molecular patterns
PD	Parkinson’s disease
PRKN	Parkin gene
PRRs	Pattern recognition receptors
PYY	Peptide YY
REM	Rapid Eye Movement
ROS	Reactive oxygen species
SCFAs	Short-chain fatty acids
SNCA	α-synuclein gene
TDC	Tyrosine decarboxylase
Th1	T helper 1 cells
Th17	T helper 17 cells
TLRs	Toll-like receptors
TNF-α	Tumor necrosis factor alpha
